# Overexpression of urokinase-type plasminogen activator in pterygia and pterygium fibroblasts

**Published:** 2011-01-06

**Authors:** Shih-Chun Chao, Dan-Ning Hu, Pei-Yu Yang, Ching-Yang Lin, Shun-Fa Yang

**Affiliations:** 1 Department of Ophthalmology, Show Chwan Memorial Hospital, Changhua, Taiwan; 2 Cell Culture Laboratory of Department of Medical Research, Show Chwan Memorial Hospital, Changhua, Taiwan; 3 National Chiao Tung University, Hsinchu, Taiwan; 4 Tissue Culture Center, New York Eye and Ear Infirmary, New York, NY; 5 Institute of Medicine, Chung Shan Medical University, Taichung, Taiwan; 6 Department of Medical Research, Chung Shan Medical University Hospital, Taichung, Taiwan

## Abstract

**Purpose:**

Urokinase-type plasminogen activator (uPA) is a protease involved in tissue remodeling and cell migration. Little is known about the expression of uPA in pterygium. The purpose of this study was to investigate the expression of *uPA* mRNA and activities in various stages of surgically excised pterygia specimens and cultured pterygium fibroblasts and to compare them with normal conjunctival tissues and fibroblasts.

**Methods:**

The expression of *uPA* mRNA and activity in 15 pterygium tissues and cultured fibroblasts from pterygium were measured using quantitative RT–PCR and zymography. Five normal conjunctiva specimens and cultured conjunctival fibroblasts were tested as the controls.

**Results:**

The expression of *uPA* mRNA and activities in pterygia and pterygium fibroblasts were significantly greater than those of the normal samples (p<0.05) and were closely related to the progression of pterygium. The amounts of *uPA* mRNA and activities in early, moderate, and advanced pterygia were 100%, 208%, and 311% and 100%, 157%, and 280% of the early stage specimens, respectively. The amounts of *uPA* mRNA and the activities in cultured pterygium fibroblasts isolated from early, moderate, and advanced pterygium specimens were 100%, 219%, and 457% and 100%, 198% and 355% of early stage fibroblasts, respectively.

**Conclusions:**

Overexpression of *uPA* was present in pterygium and their fibroblasts. The expression of *uPA* by pterygium increased significantly following the progression of the pterygium. The increased expression of *uPA* may covert plasminogen to plasmin, degrade extracellular matrixes, stimulate cell migration, induce angiogenesis, and plays an important role in the development and progression of pterygium.

## Introduction

Pterygium is a sun radiation-related common ocular surface disease that can obscure vision. It is a wing-shaped, epithelial-covered, highly proliferative and invasive fibrovascular lesion that originates from the limbus [1-16]. Compared with normal fibroblasts, pterygium fibroblasts grow much faster in a medium containing a low concentration of serum and can grow in a semisolid agar, indicating that these cells represent tumor-like transformed cells [17].

Urokinase-type plasminogen activator (uPA) is a serine protease that coverts plasminogen to plasmin and then activates pro-matrix metalloproteinases (MMPs) into MMPs; degrades various extracellular matrixes (ECM); stimulates cell migration, proliferation and chemotaxis; inhibits apoptosis; and induces angiogenesis. uPA plays an important role in tissue remolding; angiogenesis; and the progression, invasion, and metastasis of tumors [18-25].

The expression of uPA in pterygium fibroblasts and tissues has not been studied extensively [8,9]. Very little is known about the expression and secretion of uPA in pterygium cells and tissues and on the role of uPA in the pathogenesis of pterygium. To the best of our knowledge, no researchers have reported the relationship between the uPA expression and the extent/stages of pterygia.

The present study investigated *uPA* mRNA and activity levels in pterygium tissues collected from 15 patients who had pterygium removed and cultured fibroblasts isolated from 15 pterygia specimens. Five normal conjunctiva specimens and fibroblasts were tested as the controls. We studied the relationship between the expression of *uPA* in pterygia tissues and the stages of pterygium. The expression of *uPA* in cultured fibroblasts isolated from pterygia at different stages was also studied and compared with fibroblasts isolated from normal conjunctiva.

## Methods

### Subjects

All specimens were obtained from patients after surgery with the patients’ informed consent, in accordance with the tenants of the Helsinki Declaration. The present study was reviewed and approved by the Institutional Review Board of Show Chwan Memorial Hospital on Feb. 15, 2007 (code: 960102).

**Figure 1:**
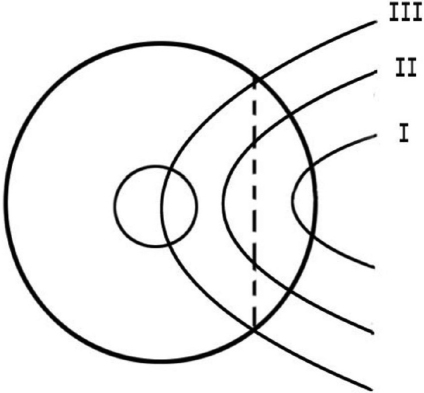
Schematic picture shows the different stages of pterygia. Stage 1: The head of pterygia did not reach the midline between the limbus and pupillary margin. Stage 2: The head of pterygia passed the midline but did not reach the pupil. Stage 3: The head of pterygia passed the pupillary margin.

The external eye of each patient was photographed before the operation. Pterygia were classified into three stages by the surgeon, based on the extent of the pterygium (Figure 1):

Stage 1: The head of pterygia did not reach the midline between the limbus and pupillary margin.
Stage 2: The head of pterygia passed the midline but did not reach the pupil.
Stage 3: The head of pterygia passed the pupillary margin.

Pterygium specimens were collected from 15 pterygium patients (7 males and 8 females, aged 62.08±12.01 years [mean±SD]). All of these pterygia were progressive in nature. The ages of the patients in stages I, II, and III were 63.75±10.21 (mean±SD), 60.4±13.15, and 62.50±15.29 years, respectively. The gender distribution (male/female) was 2/3, 3/2, and 2/3 in stage I, II, and III patients, respectively. Normal conjunctival tissues were obtained from 5 individuals (3 females and 2 males) aged 63.67±7.02 years (mean±SD).

Excised tissues were divided laterally into two identical and symmetric pieces. One piece (for measurement of tissue *uPA* mRNA and activity) was frozen and stored at −70 °C until processing. Another piece was used for the isolation and cultivation of pterygium fibroblasts.

### Isolation and cultivation of pterygium fibroblasts

Fibroblasts were obtained from 15 pterygia specimens and 5 normal conjunctival specimens (one cell line from each specimen) by using the explants methods reported by Li et al. [8] and practiced by us previously [16]. Briefly, the head of pterygium specimen or normal conjunctiva was cut into small pieces, washed, and placed into a culture dish. Dulbecco’s Modified Eagle’s Medium (DMEM) with 15% fetal bovine serum (FBS) was added to cover the explants (all from Gibco, Carlsbad, CA). The culture dish was put in a CO_2_-regulated incubator. The culture medium was replaced 3 times a week after the appearance of the outgrowth of cells from the explants. After the primary cultures became confluent, cultured fibroblasts were detached from the dish with a 0.05% trypsin/0.01% EDTA solution (Gibco, Carlsbad, CA) and passaged for subculture with a 1:3 split. In some cultures, epithelial cells also migrated from the explants in the early stage. However, epithelial cells rapidly became terminally differentiated and lost their viability in a medium with high levels of serum and Ca^2+^. Furthermore, during subculturing, the fibroblasts detached from the well much easier than the epithelial cells. After the detachment of the fibroblasts was nearly completed, serum was added to stop the effect of the enzyme so that the epithelial cells remained in the well and could not be passed onto the subculture. Therefore, only fibroblasts were present in the subcultures.

An immunocytochemical study was performed to identify the cell types. The epithelial cells contained cytokeratin antigens, but the fibroblasts did not [26].

For the uPA experiment, the fibroblasts in the second or third subcultures were seeded into a 35-mm dish and cultured with DMEM with 10% FBS. The culture was washed with PBS after near confluence and cultured with DMEM without FBS for 48 h. The conditioned medium was collected and centrifuged. The supernatant was stored at −70 °C until zymography analysis was conducted on the uPA activity. The fibroblasts were trypsinized and stored at −70 °C until *uPA* mRNA of was measured. Fifteen fibroblast cell lines from 15 pterygium specimens and 5 fibroblast cell lines from 5 normal conjunctival tissues were tested.

### Determination of uPA by casein zymography

Pterygium or normal conjunctival specimens were rinsed with PBS twice and scraped with 0.3 ml of a cold cell lysis buffer containing protease inhibitors cocktail. The protein concentrations of total lysates were determined by the Bradford assay [27].

The activities of uPA in the conditioned culture medium and pterygium tissue were measured by casein zymography protease assays, as previously described by us (Yang et al. [28]). Briefly, the cultured media or lysates of pterygium tissues were prepared with an SDS sample buffer without boiling or reduction and then subjected to 2% casein and 20 μg/ml plasminogen in 8% SDS–PAGE electrophoresis. After electrophoresis, the gels were washed with 2.5% Triton X-100 and then incubated in a reaction buffer (40 mM Tris-HCl, pH 8.0; 10 mM CaCl_2_ and 0.01% NaN_3_) at 37 °C for 12 h. Then the gels were stained with Comassie brilliant blue R-250 (all from Sigma, St. Louis, MO). The uPA standard from ELISA kits by American Diagnostica was used as the positive control and buffer was loaded as the negative control. β-actin was used as an internal loading control and analysis by western blotting. The relative photographic densities of uPA were quantitied by scanning the photographic negatives on a gel documentation and analysis system (AlphaImager 2000; Alpha Innotech Corporation, San Leandro, CA).

### RNA preparation and quantitative real-time PCR

Total RNA was isolated from tissue specimens and cultured fibroblast cells using Trizol (Life Technologies, Grand Island, NY) according to the manufacturer’s instructions. For reverse transcription, first-strand cDNA synthesis was performed with random primers (hexamers; Promega, Madison, WI) and 100 U of moloney murine leukemia virus reverse transcriptase (Promega) and performed at 42 °C for 60 min and terminated at 90 °C for 10 min. The quantitative real-time PCR analysis was performed using Taqman one-step PCR Master Mix (Applied Biosystems, Carlsbad, CA). A total of 100 ng cDNA was added per 25 μl reaction with uPA primers and Taqman probes. The *u-PA* (Hs01547054_m1) and glyceraldehyde 3-phosphate dehydrogenase (*GAPDH*; Hs99999905_m1) primers and probe were designed using commercial software (ABI PRISM Sequence Detection System; Applied Biosystems [29]). Quantitative real-time PCR assays were performed in triplicate on a StepOnePlus sequence detection system (Applied Biosystems). The cycling conditions were 10 min of polymerase activation at 95 °C followed by 40 cycles at 95 °C for 15 s and 60 °C for 60 s. The threshold was set above the nontemplate control background and within the linear phase of the target gene amplification to calculate the cycle number at which the transcript was detected.

### Statistical analysis

Statistical significances of differences throughout this study were calculated by an ANOVA one-way test in comparing data from more than two groups and using a Student’s *t*-test for comparing data between two groups. The data was analyzed using SPSS statistical software (SPSS Inc., Chicago, IL). A difference at p<0.05 was considered statistically significant.

## Results

### uPA activities in pterygium and normal conjunctival tissues

**Figure 2:**
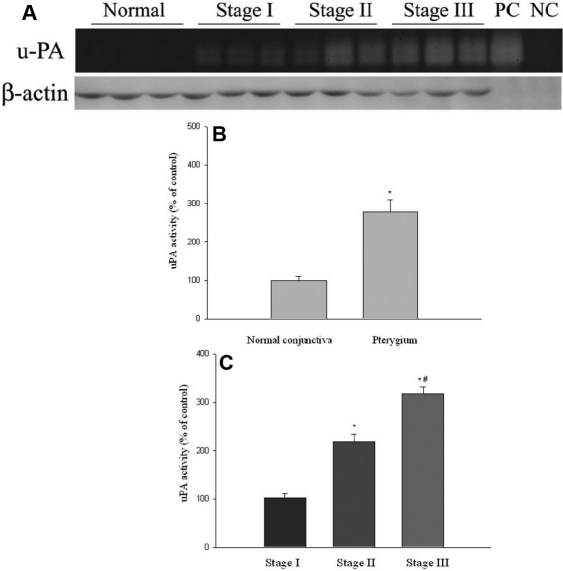
uPA activities in normal conjunctival and pterygium tissues at various stages measured by casein zymography. **A**: uPA activity was analysis by casein zymography. The u-PA standard from ELISA kits by American Diagnostica was used as the positive control (PC) and buffer was loaded alone as the negative control (NC). β-actin was used as an internal loading control and analysis by western blotting. **B**: Comparison of uPA activity levels between normal conjunctival and pterygium tissues. Data are given as mean±SD *Significant difference (p<0.05) between normal conjunctiva and pterygium. **C**: Levels of uPA in pterygium tissues at various stages. Data are given as mean±SD *Significant difference (p<0.05) compared with stage I. ^#^Significant difference (p<0.05) compared with stage II.

uPA activities in 5 normal conjunctival and 15 pterygia tissues (5 tissues from each stage) were measured by casein zymography. uPA activities in pterygium were 2.78 fold of the normal conjunctival tissues, and the difference of uPA activities between pterygium and normal tissues was statistically significantly (p<0.05; Figure 2A,B). uPA activities (expressed as percentages of stage I pterygium specimens) in stages I, II, and III pterygium tissues were 100%, 208%, and 311%, respectively. Pterygium tissues at stage III had significantly higher uPA levels than that at stages I and II (Figure 2A,C, p<0.05), the difference between stage I and stage II was also statistically significant (Figure 2A,C, p<0.05).

### *uPA* mRNA levels in pterygium and normal conjunctival tissues

**Figure 3:**
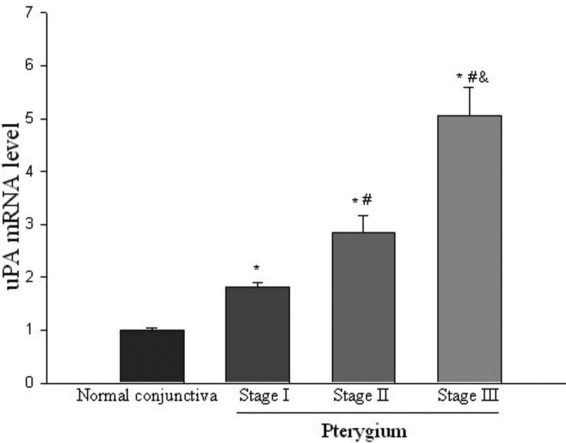
*uPA* mRNA in normal conjunctiva and pterygium tissue at various stages measured by quantitative real-time PCR. Comparison of *uPA* mRNA levels between normal conjunctiva and pterygium tissues at various stages. Data are given as mean±SD (n=5). *Significant difference (p<0.05) compared with normal. #Significant difference (p<0.05) compared with stage I. &Significant difference (p<0.05) compared with stage II.

*uPA* mRNA levels in pterygium tissues were measured by quantitative real-time PCR assay. Data from Figure 2 shows that *uPA* mRNA levels were significantly greater than those of normal conjunctival tissues. *uPA* mRNA levels in pterygium were 3.25 fold of the normal conjunctival tissues, and the difference of *uPA* mRNA levels between pterygium and normal tissues was statistically significantly (p<0.05). In pterygium specimens, *uPA* mRNA levels were expressed at various stages (Figure 3). Amounts of *uPA* mRNA (expressed as percentages of stage I pterygium specimens) in stages I, II, and III pterygium tissues were 100%, 157%, and 280%, respectively. Expression of *uPA* in stages III and II specimens was significantly greater than that of stage I specimens, the differences in *uPA* mRNA levels between stage II and III tissues was also statistically significant (p<0.05, Figure 3).

### uPA activities in conditioned media from cultured pterygium and normal conjunctival fibroblasts

**Figure 4:**
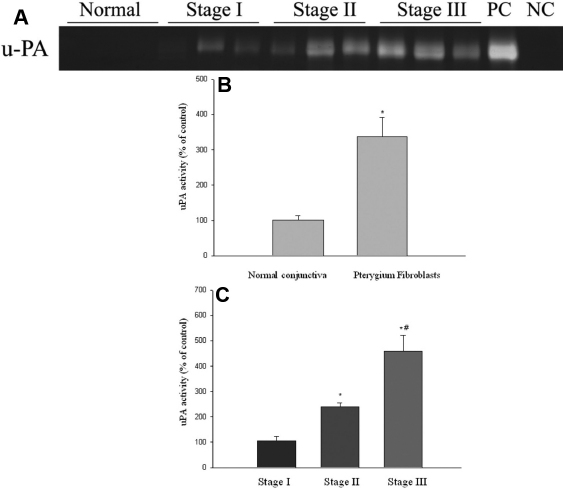
uPA activities in the conditioned media of cultured normal conjunctival and pterygium fibroblasts of various stages measured by casein zymography. Fibroblast cell lines were isolated from pterygia specimens at various stages. Normal fibroblast cell lines were isolated from normal conjunctival specimens. Cells were cultured in serum-free culture medium for 48 h. The conditioned medium was collected, and the activity of uPA was measured by casein zymography. The u-PA standard from ELISA kits by American Diagnostica was used as the positive control (PC) and buffer was loaded alone as the negative control (NC). **A**: uPA activity of medium from normal and pterygium fibroblasts was analyzed by casein zymography. **B**: Comparison of levels of uPA activity in conditioned media from normal and pterygium fibroblasts. Data are given as mean±SD *Significant difference (p<0.05) between the normal and pterygium fibroblasts. **C**: Levels of uPA activity in conditioned media of fibroblasts from various stages of pterygium. Data are given as mean±SD *Significant difference (p<0.05) compared with stage I. #Significant difference (p<0.05) compared with stage II.

uPA activity levels in conditioned media from cultured pterygium fibroblasts were 3.30 fold of the cultured normal conjunctival fibroblasts. The difference of uPA activities between pterygium and normal conjunctival fibroblasts was statistically significantly (p<0.05; Figure 4A,B). uPA activities in conditioned media from stages I, II, and III pterygium fibroblasts (expressed as percentages of stage I pterygium fibroblasts) were 100%, 219%, and 457%, respectively (Figure 4A,C). uPA activities in conditioned medium from stage III pterygium fibroblasts were significantly greater (p<0.05) than those in the media from stages I and II pterygium fibroblasts (Figure 4C). uPA levels in conditioned medium from stage II pterygium fibroblasts was also significantly greater than those from stage I pterygium fibroblasts (p<0.05, Figure 4C).

### *uPA* mRNA levels in cultured pterygium and normal conjunctival fibroblasts

**Figure 5:**
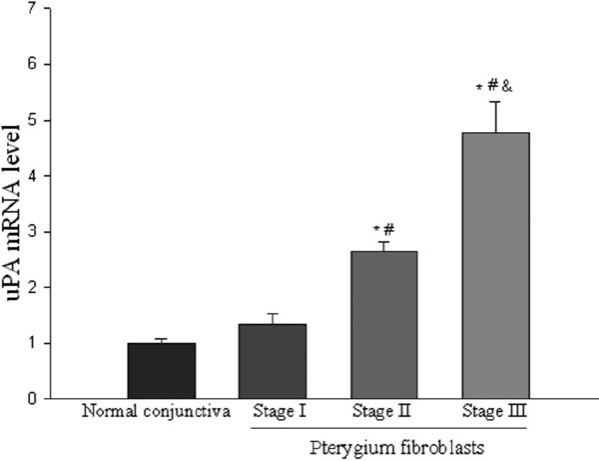
*uPA* mRNA in cultured normal conjunctival and pterygium fibroblasts of various stages measured by quantitative real-time PCR. Comparison of *uPA* mRNA levels between normal conjunctiva and pterygium fibroblasts at various stages. Data are given as mean±SD (n=5). *Significant difference (p<0.05) compared with normal. #Significant difference (p<0.05) compared with stage I. &Significant difference (p<0.05) compared with stage II.

*uPA* mRNA levels in cultured pterygium fibroblasts were 2.91 old of the cultured normal conjunctival fibroblasts. The difference of *uPA* mRNA levels between cultured pterygium fibroblasts and normal conjunctival fibroblasts were statistically significantly (p<0.05). *uPA* mRNA levels (expressed as percentages of stage I pterygium fibroblasts) in stages I, II, and III pterygium fibroblasts were 100%, 198%, and 355%, respectively (Figure 5). The difference in *uPA* mRNA levels was significant in fibroblasts between stage I and stage II (p<0.05) and between stage II and stage III (Figure 5; p<0.05).

## Discussion

Urokinase-type plasminogen activator (uPA) is a serine protease that coverts plasminogen to plasmin. It can be produced by various cells, especially tumor cells or stromal cells (e.g., fibroblasts) adjacent to a tumor [19-21,23,24,30-36]. uPA is produced as a 53 kDa single-chain protease, which is inactive and can be converted into an active two-chain uPA upon binding to the cell surface uPA receptor (uPAR) and cleavage by plasmin [18-20,22-24].

Activated uPA converts plasminogen to plasmin, a serine proteinase, which is capable of degrading fibrin. Furthermore, plasmin also degrades basement membrane and a broad spectrum of ECM, including fibronectin, vitronectin, and laminin, leading to tumor cell invasion and metastasis [19-24,37,38]. Plasmin also activates pro-matrix MMP into MMP, including MMPs-1, −2, −3, −9, −10, and 13, and results in further degradation of various ECM [22-24]. Upregulation of MMPs can be induced by binding of uPA with uPAR [21]. Many growth factors, including basic fibroblast growth factor (bFGF), vascular endothelial growth factor (VEGF), hepatocytes growth factor (HGF), insulin-like growth factor I (IGF-I), epithelial growth factor (EGF), and transforming growth factor- β (TGF- β), are activated or released from ECM by plasmin or MMP [20-24].

The binding of uPA with its receptor uPAR can activate downstream signaling molecules, including the Ras/ERK and PI3K/Akt pathways and various transcription factors, which, in turn, lead to cell proliferation, migration, and invasion [21,38,39].

uPA plays an important role in various physiologic and pathological processes, including wound healing, tissue remolding and regeneration, angiogenesis, inflammation, and tumor progression, especially in the malignant tumor invasion and metastasis [19,23,24]. Various experimental model systems have provided evidence for a causal role of uPA in cancer invasion and metastasis [19,20,24]. Numerous clinical and laboratory studies found that the uPA content in various tumor specimens is significantly increased compared with the normal tissue [18-24]. uPA is produced and released by the tumor cells or by stromal cells adjacent to a tumor [19-21,23,24,30-36]. High uPA levels in primary malignant tumor specimens correlate with a high incidence of relapse, poor prognosis, and high mortality in various tumors, including esophageal, gastric, head and neck, thyroid, breast, colorectal, liver, pancreatic, prostate, endometrial, kidney, and small cell lung cancers; chondrosarcoma, glioma, and leukemia [18-24,38]. uPA was the first proteinase shown to be a prognostic marker in human malignancies and it has remains an important prognostic marker for various human cancers [18-24,38].

Pterygium is a fibrovascular tissue from the bulbar conjunctiva and extends onto the cornea. The progression of pterygium is characterized by the degradation of corneal basement membrane and ECM, the development of angiogenesis and proliferation of fibrovascular tissue. It has been reported that the expression of several types of MMPs (MMP-1, 2, 3, 7, and 9) increases in pterygium and related cell types (fibroblasts and epithelial cells) [7-16].

Recently, we demonstrated that MMP-2 and −9 are overexpressed in pterygium tissue and fibroblasts isolated from pterygium. The levels of these MMPs in pterygium and its fibroblasts are closely related to the progression of pterygium [16]. Increased MMPs dissolve the Bowman’s layer, cause angiogenesis and invasion of pterygium onto the cornea.

MMPs are secreted in a latent precursor and can be activated by plasmin [22-24]. uPA is the most important serine proteinase that activates plasminogen into plasmin and is responsible for the tissue degradation and tumor cell invasion [18-24]. uPA plays a central role in the mediation of pericellular proteolytic activity [38]. However, little is known about the expression of uPA in pterygium.

Two papers mentioned uPA expression in pterygium as a part of their studies on the relationship between various cytokines, growth factors, MMPs with uPA and pterygium. The results were conflicting [8,9]. One paper reported the effects of several growth factors on the expression of uPA protein and various MMPs in pterygium and normal conjunctival fibroblasts. uPA released from untreated pterygium fibroblasts (approximately 0.37 pg/μg protein) seems greater than that from normal conjunctival fibroblasts (approximately 0.25 pg/μg protein), though no statistical analysis was performed by the authors [9]. Another paper measured the expression of *uPA* mRNA in pterygium by using northern blotting and stated that expression of *uPA* in pterygium did not differ from normal fibroblasts [8]. However, no statistical treatment was performed to support this conclusion. The most important parameter of various uPA levels, uPA activity that determines the ability for activated plasmin from plasminogen, has never been studied in pterygium or its cellular component, and no researchers have reported the relationship between the uPA level and the extent/stage of pterygia.

In the present study, overexpression of *uPA* mRNA and activities was present in pterygia and pterygium fibroblasts. The expression of *uPA* by pterygium and its fibroblasts is significantly increased with the progression of pterygium. The occurrence of pterygium shows similarities with tumorigenesis. During tumor development, uPA is one of the important factors involved in the growth, invasion, and metastasis [18-21,24,38]. The expression of uPA in tumor cells and stromal cells begins at the earlier stage and progressively increases during the development of tumors [18-21,24,38]. It has been hypothesized that the pterygium cells are tumorlike altered limbal fibroblasts and epithelial cells [7,17]. Pterygium fibroblasts grow well in a culture medium with only a low concentration of serum, and they can grow in a semisolid sugar, indicative of anchorage-independent growth, which is a phenotype of transformed or tumor cells [17]. The present study demonstrated that fibroblasts from pterygium produce and release higher levels of uPA compared with normal conjunctival fibroblasts. Cells from advanced stages of pterygia produce and release more uPA than the early-stage pterygium fibroblasts. This is consistent with the findings that fibroblasts adjacent to the tumor overexpress *uPA* and play a role in the tumor invasion and progression [19-21,24,30-36].

Understanding of the role of uPA in the development of pterygium has relevance to devising a new strategy of pterygium treatment [24,37-40]. The involvement of the uPA system in cancer progression and the observation that its inhibition is devoid of toxicity, as demonstrated in uPA-deficient mice, identifies the uPA system as a suitable target for anti-cancer therapies [24,37-40]. Several therapeutic strategies inhibiting the inhibition of the uPA system have been or are being developed for suppression of tumor growth and progression, including anti-uPA antibodies, small molecular uPA inhibitors, cytotoxins targeting uPA/uPAR components, antisense therapy, and RNA interference technology [24,37-40]. Various selective uPA inhibitors have shown promising results in experimental animal studies and preclinical testing [24,37-40]. Some of these therapies have been proven to be safe and well tolerated in Phase I clinical studies and have entered Phase II clinical studies [24]. Therefore, further studies in this field might be helpful for the development of novel therapies in the management of pterygium and in the prevention of its relapse.
